# Contact Urticaria and Related Conditions: Clinical Review

**DOI:** 10.1111/cod.14794

**Published:** 2025-04-02

**Authors:** Mojca Bizjak, Olivier Aerts, David Pesqué, Melba Muñoz, Riccardo Asero, Margarida Gonçalo, Thomas Rustemeyer, Mitja Košnik, Mark Kačar, An Goossens, Jose Hernán Alfonso, Charlotte G. Mortz, Maryam Ali Al‐Nesf, Joachim W. Fluhr, Howard I. Maibach, Ana Maria Giménez‐Arnau

**Affiliations:** ^1^ Division of Allergy University Clinic of Respiratory and Allergic Diseases Golnik Golnik Slovenia; ^2^ Faculty of Medicine, University of Maribor Maribor Slovenia; ^3^ Department of Dermatology Antwerp Belgium; ^4^ Dermatology Department, Hospital del Mar Research Institute, Department of Medicine Universitat Autònoma de Barcelona (UAB) Barcelona Spain; ^5^ Institute of Allergology, Charité – Universitätsmedizin Berlin, corporate member of Freie Universität Berlin, Humboldt‐Universität zu Berlin, and Berlin Institute of Health Berlin Germany; ^6^ Fraunhofer Institute for Translational Medicine and Pharmacology ITMP Immunology and Allergology Berlin Germany; ^7^ Clinica San Carlo Ambulatorio di Allergologia Milano Italy; ^8^ Clinic of Dermatology University Hospital, Coimbra Local Health Unit and Faculty of Medicine, University of Coimbra Coimbra Portugal; ^9^ Dermato‐Allergology and Occupational Dermatology Amsterdam University Medical Centers Amsterdam the Netherlands; ^10^ Faculty of Medicine, University of Ljubljana Ljubljana Slovenia; ^11^ Faculty of Medicine Catholic University of Leuven (KU Leuven) Leuven Belgium; ^12^ Department of Dermatology & Venereology Oslo University Hospital – Rikshospitalet Oslo Norway; ^13^ Department of Occupational Medicine and Epidemiology National Institute of Occupational Health Oslo Norway; ^14^ Department of Dermatology and Allergy Centre Odense University Hospital, University of Southern Denmark Odense Denmark; ^15^ Allergy and Immunology Division, Department of Medicine Hamad Medical Corporation Doha Qatar; ^16^ Department of Dermatology University of California San Francisco San Francisco California USA; ^17^ Dermatology Department Hospital del Mar Research Institute, Universitat Pompeu Fabra (UPF) Barcelona Spain

**Keywords:** anaphylaxis, contact urticaria, inducible urticaria, occupational urticaria, protein contact dermatitis

## Abstract

Contact urticaria (CoU) is an immediate contact reaction occurring within minutes to an hour after exposure to specific proteins or chemicals. CoU is categorised into non‐immunologic (NI‐CoU) and immunologic (I‐CoU) types, with I‐CoU potentially leading to anaphylaxis. Both forms of CoU can be associated with protein contact dermatitis and the CoU syndrome. Patients with I‐CoU may also have other type I (immediate) allergic diseases, such as allergic conjunctivitis, rhinitis, asthma or food allergy. This review provides a detailed overview of CoU and related conditions, focusing on triggers, diagnostic methods and management strategies. NI‐CoU is typically triggered by low molecular weight chemicals, while I‐CoU involves IgE‐mediated hypersensitivity to both high molecular weight proteins and low molecular weight chemicals. Early diagnosis is crucial, though CoU is often underrecognized. The diagnostic approach includes a thorough medical history, physical examination, evaluation of photographs, (non)invasive skin tests and in vitro assessments. Management strategies prioritise trigger avoidance and pharmacological treatments when avoidance is not fully possible. For I‐CoU, second‐generation H_1_‐antihistamines are the first‐line treatment. Severe cases of I‐CoU may benefit from anti‐IgE therapy (omalizumab). Patients at risk of anaphylaxis should carry an adrenaline auto‐injector and wear a medical alert bracelet.

**Abbreviations**
CoU: contact urticaria
HMW: high molecular weight
I‐CoU: immunologic contact urticaria
ICR(s): immediate contact reaction(s)
IgE, IgG, IgM: immunoglobulin E, G, M
LC: Langerhans cells
LMW: low molecular weight
NI‐CoU: non‐immunologic contact urticaria
NI‐ICR(s): non‐immunologic immediate contact reaction(s)
PCD: protein contact dermatitis
sgAH(s): second‐generation H_1_‐antihistamine(s)
SPT(s): skin prick test(s)

## 1 Introduction

Contact urticaria (CoU), a subtype of inducible urticaria, is an immediate contact reaction (ICR) that occurs within minutes to an hour following contact with specific chemicals [1, 2]. These chemicals can be categorised as low molecular weight (LMW, < 1000 Da [g/mol]) and high molecular weight (HMW, ≥ 1000 Da). CoU is classified into two types: non‐immunologic (NI‐CoU) and immunologic (I‐CoU). NI‐CoU is regarded as an irritant reaction, while I‐CoU represents a type I (immediate) hypersensitivity, which can progress to anaphylaxis [1]. Patients with I‐CoU may also experience other type I allergic diseases, such as allergic conjunctivitis, rhinitis or asthma (Table 1) [6, 7, 8, 9, 10, 11, 12, 13, 14, 15]. Additionally, both NI‐CoU and I‐CoU may be associated with protein contact dermatitis (PCD) and are part of the CoU syndrome [1]. Differentiating between NI‐CoU and I‐CoU can be challenging, especially with non‐protein substances. However, making this distinction is important as it guides the most effective treatment approach [1, 16].

**TABLE 1:** Clinical manifestations of type I allergic responses by exposure site.

Exposure	Diagnosis	Possible symptoms and signs
Skin (topical)	Immunologic contact urticaria (I‐CoU)	Itching, burning, tingling, stinging, erythema, localised or generalised wheals, angioedema
	Anaphylaxis [3, 4, 5]	Skin/visible mucosa: flushing/erythema, itching, wheals, angioedema Respiratory system: dysphagia, dysphonia, dyspnea, wheeze, stridor, sneezing, cough, chest pain Gastrointestinal system: crampy abdominal pain, vomiting, diarrhoea Cardiovascular system and associated organs: dizziness, lightheadedness, confusion, hypotonia, weakness, collapse, syncope, tachycardia, blood pressure < 90/60 mmHg
Conjunctival (topical)	Conjunctivitis	Itchy, watery eyes; burning, hyperemia, swollen eyelids
	Anaphylaxis	See above
Respiratory (inhalation)	Allergic rhinitis	Runny/itchy nose, nasal obstruction, sneezing
	Allergic asthma	Shortness of breath, wheezing, chest tightness, coughing, tachypnea
	Anaphylaxis	See above
Oral (ingestion)	Oral allergy syndrome	Itching, burning; angioedema (lips, tongue, roof of the mouth or throat)
	Anaphylaxis	See above

The epidemiology of CoU remains poorly documented, but both NI‐CoU and I‐CoU appear to be relatively common [16]. A Finnish survey involving 2759 patients with occupational allergic contact dermatoses found that 29.5% of the cases were CoU and 70.5% allergic contact dermatitis [17]. In contrast, a German study involving 171 053 dermatology and allergy patients reported a CoU frequency of less than 0.4% [6]. Differences in the occurrence of CoU among countries may be due to underreporting [18]. Occupations at higher risk for CoU include healthcare workers, laboratory workers, cooks, bakers, butchers, restaurant personnel, veterinarians, hairdressers, florists, gardeners and forestry workers [7, 18, 19, 20].

The wheals associated with CoU resemble those seen in other forms of urticaria, but their shape can vary depending on the type of contact. For example, stinging nettle ( *Urtica dioica* ) may produce linear wheals, while follicular patterns can occur when agents penetrate hair follicles. In more severe cases, reactions may result in confluent wheals [21]. In I‐CoU, the cutaneous triple response comprising itching, whealing and flare [22] is expected.

The aim of this clinical review is to provide a detailed overview of CoU and its associated systemic and eczematous manifestations, focusing on clinical presentations, triggers, diagnostic methods and management strategies.

## 2 Clinical Presentations and Triggers

### 2.1 Non‐Immunologic Immediate Contact Reactions

Non‐immunologic ICRs (NI‐ICRs) typically present with a burning sensation and erythema, often without true whealing [23, 24, 25]. When whealing occurs, the term NI‐CoU can be used (Table 2). The triggers are primarily LMW, non‐protein chemicals such as benzoic acid [60, 61, 62, 68, 69, 70, 71, 72, 73], cinnamic acid [60, 61, 62, 71], cinnamic aldehyde (cinnamal) [6, 23, 60, 61, 62, 63, 67, 114], dimethyl sulfoxide [62, 71, 72], nicotinic acid esters [25, 71, 72, 73], sodium benzoate [62, 224, 225] and sorbic acid (Table 3) [6, 61, 62, 68, 115]. NI‐ICRs can occur upon first contact with triggering substances and are dose‐dependent. Wheals may develop within minutes to an hour after exposure; although, in rare cases, onset may be delayed for up to 6 h [23]. Typically, NI‐ICRs remain localised to the contact area and resolve within minutes to hours upon avoidance, usually completely within 24 h [1]. Burning, stinging, and/or tingling sensations are more prominent than pruritus (Table 2).

**TABLE 2:** Comparison of non‐immunologic and immunologic contact urticaria.

	Non‐immunologic contact urticaria (NI‐CoU)	Immunologic contact urticaria (I‐CoU)
Prevalence	Frequent	Relatively rare; more common in atopic individuals [26, 27]
Culprits	Mainly LMW chemicals Proteins < other chemicals	Mainly HMW, occasionally LMW chemicals Proteins > other chemicals
	Examples: benzoic acid, cinnamic acid, cinnamic aldehyde (cinnamal), dimethyl sulfoxide, nicotinic acid esters, sodium benzoate and sorbic acid (Table 3)	Examples: cosmetic and industrial chemicals, food additives, plant and animal products, pharmaceuticals (Table 3)
Mechanism	Irritant	IgE‐mediated
Medical history	Erythema, edema (whealing) – onset within a few minutes to an hour after exposure (usually slower than I‐CoU) – only erythema may occur – rarely spreads beyond contact sites – disappears within hours, 24 h at the latest	Erythema, edema (whealing) – onset within a few minutes to an hour after exposure (usually faster than NI‐CoU) – may spread beyond contact site, anaphylaxis may occur – disappears within hours, 24 h at the latest
	Burning, stinging and/or tingling > pruritus	Pruritus > burning, stinging and/or tingling
Protein contact dermatitis (PCD)	It may occur with repeated exposure – Irritant	It may occur with repeated exposure – A mix of type I (immediate, IgE‐mediated) and IV (delayed, T cell‐mediated) hypersensitivity^a^ – ‘Pure’ type I (immediate) hypersensitivity
Diagnostic tests	*Non‐invasive skin test* ^b^ Open test Chamber test	*Non‐invasive skin test* ^b^ Open test Chamber test *Invasive skin tests* ^c^ Skin prick Prick‐by‐prick Intradermal In vitro *techniques* ^d^ Antigen‐specific serum IgE antibodies Basophil activation test, histamine release test
Diagnostic criteria	Suggestive medical history Suggestive photographs of skin lesions (if available) Positive non‐invasive skin test	Suggestive medical history Suggestive photographs of skin lesions (if available) Positive non‐invasive skin test Confirmed immediate‐type sensitization by means of SPTs and/or specific IgE tests^d^ and established clinical relevance based on exposure history
Management	Complete avoidance or attempt to ↓ exposure/dose	Complete avoidance sgAHs if complete avoidance is difficult Omalizumab (also for I‐CoU‐related PCD) Dupilumab for PCD Adrenaline auto‐injector if indicated

Abbreviations: HMW, high molecular weight; I‐CoU, immunologic contact urticaria; LMW, low molecular weight; NI‐CoU, non‐immunologic contact urticaria.

^a^ Most authors claim the co‐occurrence of type I and type IV hypersensitivity to the same substance.

^b^ Non‐invasive skin tests are usually performed sequentially, with each subsequent test considered only if the previous one is negative.

^c^ Invasive skin tests are only performed on unaffected skin. Some dermatologists routinely use SPTs and/or prick‐by‐prick tests as a first method for detecting I‐CoU.

^d^ In case of a history of systemic signs/symptoms, in vitro methods should be considered as the first step if available.

**TABLE 3:** Triggers of contact urticaria and related conditions.

Trigger	Contact urticaria (CoU)	Immunologic contact urticaria (I‐CoU)^a^	Contact‐induced anaphylaxis^b^	Conjunctivitis, rhinitis, asthma^c^	Main sources (functions)
Algae	[28]	[28]		[28]	Cosm., food
***p*‐Aminophenol** ^d^, ^e^	[29, 30, 31]	[29, 30, 31]	[29, 30, 31]		Cosm. (HDF)
**Ammonium persulphate** ^d^, ^e^	[13, 32, 33, 34, 35, 36, 37]	[33, 34]	[34, 35, 36, 38]	[34, 35, 39, 40]	Cosm. (HDF)
α‐amylase^f^, ^g^	[17, 41, 42]	[41, 42]		[41, 43]	Food, textile ind. (ENZ)
Apple^f^, ^h^	[44, 45]	[45]			Food
Asparagus^f^	[46, 47]	[46, 47]		[47, 48, 49]	Food
Bacitracin^e^	[50, 51, 52, 53, 54, 55]	[50, 51, 52, 53, 54, 55]	[50, 51, 52, 53, 54, 55, 56, 57, 58, 59]		Pharm. (AB)
Balsam of Peru ( *Myroxylon pereirae* )^d^, ^e^, ^i^	[6, 24, 60, 61, 62, 63, 64, 65, 66]				Cosm., food, pharm. (FR/FL)
**Benzaldehyde** ^d^, ^e^	[6, 60, 67]				Cosm., clean., food (FR/FL)
**Benzoic acid** ^d^, ^e^, ^i^	[60, 61, 62, 68, 69, 70, 71, 72, 73]				Cosm., clean., food (PR/AM)
**Benzophenone‐3** ^d^, ^e^ **(oxybenzone)**	[74, 75, 76, 77, 78]	[74]	[74, 75, 76]		Cosm. (UV‐adsorber)
Bisphenol A epoxy resin^e^	[79]	[79]			Ind. (adhesives, coatings)
Cannabis^d^	[80, 81]	[81]		[80]	Cosm.
**Capsaicin** ^d^	[22]				Cosm., pharm. (analgetics)
Carrot^f^, ^h^	[44, 82, 83, 84]	[82, 83, 84]		[82, 83, 84]	Food
**Carvone** ^d^	[85]			[85]	Cosm., food (FR/FL)
Caterpillars	[86, 87, 88, 89]	[88, 89]	[88, 89]	[88]	Animal origin
Cellulase^g^	[17, 90, 91, 92]	[90, 91]		[90, 91]	Food, paper ind. (ENZ)
Cefotiam	[93, 94]	[94]	[94]	[94]	Pharm. (AB)
Chamomile ( *Chamomilla recutita* )^d^	[95, 96]	[95, 96]		[95]	Cosm.
Chlorhexidine^d^, ^e^, ^f^, ^j^	[97, 98, 99, 100, 101, 102]	[97, 98, 99, 101, 102]	[97, 98, 99, 100, 101, 102, 103, 104, 105, 106]	[107]	Cosm., pharm. (PR/AM)
**Chlorocresol (*p*‐Chloro‐m‐cresol)** ^d^, ^e^	[108, 109, 110]			[109]	Cosm., pharm. (PR/AM)
**Chromium (potassium dichromate)** ^e^	[24]				Metals
Chrysanthemum^h^	[111, 112, 113]				Inedible plants
**Cinnamic acid** ^d^, ^i^	[60, 61, 62, 71]				Cosm., food (FR/FL)
**Cinnamic aldehyde (cinnamal)** ^d^, ^e^, ^k^	[6, 23, 60, 61, 62, 63, 67, 114]				Cosm., food (FR/FL)
*Cinnamomum cassia* oil^d^	[64, 115]				Cosm. (FR)
**Cobalt (cobalt chloride)** ^e^	[24, 62]				Metals
Colophonium^d^, ^e^	[24]			[116]	Cosm., ind. (adhesives)
**Copper** ^e^	[117]	[117]		[117]	Metals
**Coumarin** ^d^, ^e^, ^l^	[1]				Cosm., clean., food (FR/FL)
Cow's dander^f^, ^h^	[6, 8, 92, 118]	[6, 118]		[8, 92, 118]	Animal origin
Cow's milk^f^, ^h^	[27, 119, 120, 121]	[27, 119–121]			Food
**Dimethyl sulfoxide**	[62, 71, 72]				Pharm. (solvents)
**Diphenylmethane‐diisocyanate**	[122]	[122]		[92, 122]	Ind. (foams, paints)
Egg^f^	[6, 17, 123]	[6, 123]		[11]	Food
Equae Lac^d^ (mare's milk^f^)	[124, 125]	[124, 125]			Cosm. (skin care)
**Ethyl alcohol** ^d^ **(ethanol)**	[62, 126]	[126]			Cosm., food, pharm. (PR/AM)
**2‐ethylhexyl acrylate** ^d^, ^e^	[17, 92]				Cosm., ind. (adhesives, coatings)
**Eugenol** ^d^, ^e^, ^k^	[110, 127, 128]	[127]		[129]	Cosm., pharm. (FR/FL)
*Ficus benjamina* ^f^	[112, 130]	[112, 130]		[130]	Inedible plants
Fishing baits (worms)	[131, 132]	[131, 132]		[132]	Animal origin
**Formaldehyde** ^d^, ^e^, ^f^	[24, 133]	[133]	[133, 134]	[11, 135]	Cosm., clean., ind. (PR/AM)
Garlic^f^, ^h^	[136]	[136]		[11, 137, 138]	Food
**Geraniol** ^d^, ^e^, ^k^	[139]				Cosm. (FR)
**Glycolic acid** ^d^	[140]				Cosm. (exfoliation)
Honey (Mel)^d^, ^f^	[141, 142]	[141]			Cosm. (EM, AM)
Hydolysed animal proteins (h. collagen^d^, h. milk protein^d^)	[143, 144, 145, 146]	[143, 144, 145, 146]	[143, 144]	[144]	Cosm., food (ES)
Hydrolysed wheat protein^d^	[147, 148, 149, 150, 151, 152, 153]	[147, 148, 149, 150, 151, 152, 153]		[147, 150]	Cosm., food (ES)
Jellyfish venom	[62, 154, 155]	[154, 155]	[154, 155]		Animal origin
Kiwi^f^	[156]				Food
Lanolin alcohol^d^, ^e^	[24]				Cosm., pharm. (EM)
Lilies (*Lilium*)	[19, 157]	[19, 157]		[19, 157]	Inedible plants
Mango^f^, ^h^	[6, 158]	[6, 158]			Food
Meat, beef^f^, ^h^	[159, 160, 161]	[159, 160, 161]			Food
Meat, pork^f^, ^h^	[17]				Food
Meat, chicken^f^, ^h^	[17]				Food
**Menthol** ^d^, ^e^, ^m^	[162]	[162]	[162]	[163, 164, 165]	Cosm., food, pharm. (FR/FL)
**Methylisothiazolinone** ^d^/**methylchloroisothiazolinone** ^d^, ^e^	[24]				Cosm., clean., ind., pharm. (PR)
Natural rubber latex (NRL)^f^, ^h^	[6, 8, 17, 92, 166, 167, 168]	[6, 166, 167, 168]	[6, 106, 166, 167]	[8, 11]	Plants
**Neomycin (neomycin sulphate**)^e^	[24]		[52, 169]		Pharm. (AB)
**Nickel (nickel sulphate)** ^e^	[24, 170, 171, 172]				Metals
Nicotinic acid esters (e.g., **methyl nicotinate**)^d^	[25, 71, 72, 73]				Cosm., pharm.
Oat ( *Avena sativa* )^d^, ^f^, ^h^	[173, 174]	[173, 174]			Cosm., food
Onion^f^, ^h^	[6, 17, 44, 92]	[6]			Food
**Panthenol** ^d^, ^e^	[175]	[175]			Cosm. (EM)
Papain^f^, ^g^	[176, 177, 178]	[176, 177, 178]		[179, 180]	Cosm., food, pharm. (ENZ)
**Paraben(s)** ^d^, ^e^	[24, 181]				Cosm., food, pharm. (PR/AM)
**Paratoluenediamine (PTD, toluene‐2,5‐diamine sulphate)** ^d^, ^e^	[182]	[182]	[182]		Cosm. (HDF)
Peach^f^, ^h^	[183, 184]	[183, 184]			Food
**Phenoxyethanol** ^d^, ^e^	[185, 186, 187, 188, 189, 190, 191, 192, 193]	[186]	[186]		Cosm., pharm. (PR/AM)
***p*‐Phenylenediamine** ^d^, ^e^	[24, 194, 195, 196, 197]	[194]	[194]	[197]	Cosm. (HDF), ind.
Polyhexanide (polyaminopropyl biguanide)^d^, ^e^, ^f^, ^j^	[198, 199]	[198, 199]	[198, 199]		Cosm., pharm., ind. (PR/AM)
Polymyxin B^e^	[200, 201]	[200, 201]	[200, 201]		Pharm. (AB)
Potato^f^, ^h^	[6, 44, 202]	[6, 202]		[202]	Food
**Potassium persulphate** ^d^	[17, 203]	[203]			Cosm. (HDF), ind.
Povidone (PVP, polyvinylpyrrolidone)^d^, ^e^	[204, 205, 206, 207]	[204, 205, 206, 207]	[204, 205, 206, 207]		Cosm., pharm. (binders, stabilisers)
Povidone iodine^e^	[208]	[208]	[106, 209]		Pharm. (AM)
**Pramoxine** ^e^	[210]	[210]	[210]		Pharm. (anaesthetic, antipruritic)
Protease^g^	[211]	[211]		[11, 211]	Food, pharm., clean. (ENZ)
Rice^f^, ^h^	[212]	[212]		[212]	Food
Runner bean ( *Phaseolus multiflorus* )	[213]	[213]			Food
Rye^f^, ^h^	[214]	[214]		[11, 214]	Food
Salmon^f^, ^h^	[160]	[160]			Food
Seafood (i.e., fish, crustaceans)^f^, ^h^	[8, 160, 215, 216]	[160, 215, 216]		[11, 217]	Food
Sesame ( *Sesamum indicum* )^d^, ^f^, ^h^	[218]	[218]			Cosm., food
Silk^f^	[219, 220, 221]	[219, 220]	[219]	[222, 223]	Animal origin
**Sodium benzoate** ^d^, ^e^	[62, 224, 225]				Cosm., clean., food, pharm. (PR/AM)
**Sodium hypochlorite**	[1, 226]	[1]	[226]		Clean., ind. (AM)
**Sorbic acid** ^d^, ^e^	[6, 61, 62, 68, 115]				Cosm., clean., food, pharm. (PR/AM)
Soybean^f^, ^h^	[136]	[136]		[136]	Food
Spider mite (*Tetranychus urticae*)	[227]	[227]		[11, 227]	Animal origin
Spices	[8, 17]			[11]	Food
Stinging nettle ( *Urtica dioica* )^f^, ^h^	[228]				Plants
Storage mites^f^	[17]			[92]	Animal origin
Streptomycin^e^	[229]				Pharm. (AB)
Tomato^f^, ^h^	[44]				Food
**Triclosan** ^d^, ^e^	[230]	[230]	[230]		Cosm., clean., pharm. (PR/AM)
Tulip	[19, 157]	[19, 157]		[19, 157]	Inedible plants
**Turpentine** ^d^, ^e^	[1]			[231]	Cosm. (FR)
**Vanillin** ^i^	[1, 64]				Cosm. (FR)
Walnut^f^, ^h^	[232]	[232]			Food
Wheat^f^, ^h^	[92, 214]	[92, 214]		[92, 214]	Food
Wool (ewe)	[233]				Animal origin
Xylanase (hemicellulase)^g^	[90, 234]	[90, 234]		[11, 90, 234]	Paper ind. (ENZ)

*Note*: Low molecular weight (LMW) chemicals (< 1000 Da [g/mol]) are marked in bold. Data on Chemical Abstracts Service (CAS) registry numbers of triggers can be found in Table S1.

Abbreviations: AB, antibiotics; AM, antimicrobials; Clean., cleaning products; Cosm., cosmetics; CoU, contact urticaria; EM, emollients/moisturisers; ENZ, enzymes; ES, emulsifiers/stabilisers; FL, flavourings; FR, fragrances; HDF, hair dye formulations; I‐CoU, apparently immunologic contact urticaria; Ind., industry/industrial use; Pharm., pharmaceuticals; PR, preservatives.

^a^ In many cited cases, the question of whether CoU was IgE‐mediated or not remained slightly open.

^b^ Induced from topical contact or airborne fumes/particles, not via ingestion.

^c^ Due to exposure to the fumes/particles in the air (usually occupational).

^d^ INCI (International Nomenclature of Cosmetic Ingredients) name.

^e^ Commercially available for patch testing.

^f^ Commercially available for CAP testing.

^g^ α‐amylase, cellulase, papain, protease and xylanase (hemicellulase) are industrial enzymes.

^h^ Commercially available for skin prick testing.

^i^ Benzoic acid, cinnamic acid and vanillin may also be present in balsam of Peru ( *Myroxylon pereirae* ) resin [235].

^j^ Chlorhexidine and polyhexanide share the hexamethylene biguanide [199].

^k^ Cinnamic aldehyde (cinnamal), eugenol and geraniol are also present in *Fragrance mix I* commercially available for patch testing.

^l^ Coumarin is also present in *Fragrance mix II* commercially available for patch testing.

^m^ Menthol is a cyclic alcohol derived from mint plants—peppermint (*Mentha piperita* ^f^) and spearmint ( *Mentha spicata* ) [162, 164].

The exact mechanisms underlying NI‐ICRs are not fully understood [23, 24]. These reactions may involve a direct effect of exogenous substances on dermal blood vessels or nerves. Additionally, the non‐antibody‐mediated release of histamine, prostaglandins, leukotrienes (from mast cells and other immune cells) and substance P (from sensory neurons) may contribute to the vasodilation [69, 185, 236].

### 2.2 Immunologic Contact Urticaria

I‐CoU is an immunoglobulin E (IgE)‐mediated hypersensitivity reaction requiring prior sensitization, which can occur through the skin, respiratory system or gastrointestinal tract. Upon re‐exposure, allergens penetrate the epidermis (often through a disrupted skin barrier), bind to IgE and trigger histamine release from mast cells. Lipophilic allergens may also enter through hair follicles [237]. I‐CoU is induced by HMW or LMW chemicals, including cosmetic and industrial chemicals, food additives, plants or plant products, animals or animal products and pharmaceuticals (Table 3) [2, 238]. Among healthcare workers, natural rubber latex (NLR) was once a common trigger of I‐CoU, but its incidence has declined due to the increased use of synthetic rubber gloves [1, 32]. LMW chemicals can induce IgE‐mediated sensitization, but must first bind to carrier proteins, such as serum albumin, to form hapten‐protein conjugates [8, 239]. Examples include diphenylmethane‐diisocyanate [122] and p‐phenylenediamine (Table 3) [194]. I‐CoU caused by ammonium persulphate can be mediated by antigen‐specific IgE [33], immunoglobulin G (IgG) or immunoglobulin M (IgM) [21, 33, 240].

Wheals typically appear within minutes to an hour, faster than in NI‐CoU [1, 21, 26, 239, 241]. Pruritus is more prominent than burning, stinging and/or tingling sensations, and wheals usually resolve within hours, typically by 24 h after avoidance [1]. However, I‐CoU can sometimes have a delayed onset after repeated exposure [1, 21, 242], possibly due to slower penetration [93, 242].

### 2.3 Oral Allergy Syndrome

Oral allergy syndrome is a type of I‐CoU that may present as itching, burning, and in some cases, swelling of the lips, tongue, roof of the mouth or throat within minutes of ingesting the allergen [243]. This is a frequent manifestation of food allergy in adults. It may occur not only in individuals with the so‐called pollen‐food syndrome (which is caused by the cross‐reactivity between pollen allergens, such as the major birch allergen [Bet v 1] or profilin [a plant panallergen] with homologous, heat‐ and pepsin‐sensitive allergens in various fruits, vegetables and nuts) [15, 239], but also in those sensitised to heat‐ and pepsin‐stable food allergens, such as lipid transfer protein found in many plant‐based foods [244].

### 2.4 IgE‐Mediated Contact‐Induced Anaphylaxis

Anaphylaxis induced through topical exposure (i.e., contact‐induced anaphylaxis), rather than by ingestion, is primarily triggered by proteins, although some LMW chemicals can also be responsible. This type of anaphylaxis is believed to occur when substances provoke a strong hypersensitivity reaction or are readily absorbed due to their LMW and optimal solubility [224, 245]. Additionally, many reported cases involved patients applying triggering agents to compromised skin barriers (e.g., excoriated dermatitis, abrasions) or to mucosal surfaces without a stratum corneum (e.g., during urological surgery [103]), which facilitated greater access to the systemic circulation [56, 57]. Another contributing factor could be the application of allergens to large areas of skin (e.g., sunscreen containing benzophenone‐3) [74, 75, 76].

Contact‐induced anaphylaxis has been mainly reported in association with substances found in hair dye formulations (e.g., p‐aminophenol [29, 30, 31], ammonium persulphate [34, 35, 36, 38], paratoluenediamine [182], p‐phenylenediamine [194]), antibiotics (e.g., bacitracin [50, 51, 52, 53, 54, 55, 56, 57, 58, 59], cefotiam [94], neomycin [52, 169], polymyxin B [200, 201]), preservatives and antimicrobials (e.g., chlorhexidine [97, 98, 99, 100, 101, 102, 103, 104, 105, 106], formaldehyde [133, 134], phenoxyethanol [186], polyhexanide [198, 199]), hydrolysed animal proteins [143, 144], jellyfish venom [154, 155], menthol [162], NLR [6, 106, 166, 167], povidone [204, 205, 206, 207], pramoxine [210] and silk [219] (Table 3).

### 2.5 Protein Contact Dermatitis

PCD is a localised eczema/dermatitis that can be associated with both I‐CoU and NI‐CoU [1]. It is typically, though not exclusively, occupational in nature [158, 246]. The exact mechanism of PCD is not fully understood. Most authors claim the co‐occurrence of type I (immediate, IgE‐mediated) and type IV (delayed‐type, T‐cell‐mediated) hypersensitivity reactions [247] to the same substance [1, 158, 241]. In 1988, Bruynzeel‐Koomen et al. demonstrated that epidermal Langerhans cells (LC) from patients with atopic dermatitis bind IgE molecules via their Fc fragment [248]. In 1992, Bieber et al. showed that human epidermal LC express the high affinity receptor for IgE [249]. In the same year, it was suggested that LC likely play a role in transepidermal, IgE‐mediated allergy [250]. An allergen bound to IgE on dermal mast cells can induce an immediate reaction, while the same allergen bound to IgE on LC may facilitate antigen presentation to T lymphocytes, leading to a delayed reaction [136, 251].

The acute phase of PCD occurs within minutes and is characterised by an initial ICR (i.e., erythema and/or whealing), which may not be obvious to patients. In the subacute phase, (micro)vesicles may be present, while more chronic forms of PCD may present with lichenification, fissures, scaling and excoriations. PCD tends to affect the hands (i.e., chronic hand eczema), particularly the fingertips (i.e., pulpitis and chronic paronychia) [246]. In a retrospective study, Azevedo et al. demonstrated that among 52 patients with chronic hand eczema experiencing immediate symptoms, 54% had positive skin prick tests (SPTs) with suspected substances, most commonly NLR and kiwi [252]. However, some latex allergens cross‐react with plant‐derived food allergens (e.g., kiwi, banana, avocado) [253]. Additionally, topical exposure to aeroallergens (e.g., grass pollen proteins) in sensitised patients with atopic dermatitis may induce eczema flare‐ups [254]. This reaction could be considered a subtype of PCD, an under‐recognised phenomenon.

## 3 Diagnostic Workup

A critical part of the diagnostic workup of CoU and related conditions is obtaining a thorough medical history with a high degree of suspicion. Patients should be asked about their symptoms and signs (Tables 1 and 2), the time from exposure to onset, the duration until resolution and potential triggers such as food, workplace exposures, personal care products, habits and medications. It is important to determine whether symptoms began before or after starting a particular job and if there is improvement during time away from work. Collaboration with occupational physicians is often necessary to assess potential triggers [255]. In patients with CoU, skin reactions are often absent during physical examination, making the evaluation of photographs of skin lesions particularly valuable.

Diagnostic tests must be carefully selected, performed and interpreted by trained professionals. False‐positive results can lead to unnecessarily restrictive measures and negatively impact a patient's quality of life, while false‐negative results may result in continued exposure to triggering substances. Skin tests are summarised in Table 4.

**TABLE 4:** Skin tests for diagnosing immediate contact skin reactions.

Skin test	Procedure	Reading time	Positive result criteria^a^
Open	Apply liquid (0.1 mL) or solid material over 3 × 3 cm skin area without occlusion for 15–20 min. For multiple liquid substances, apply 10 μL on 1 × 1 cm skin areas.	15–20 min, +20 min, +20 min	Whealing^b^ Erythema^c^
Rub	Gently rub solid material over 3 × 3 cm skin area 10 times, leave material on skin for 15–20 min.	15–20 min, +20 min, +20 min	Whealing^b^ Erythema^c^
Chamber	Place material into a chamber, apply to skin for 15–20 min.	5 min after removal, +20 min, +20 min	Whealing^b^ Erythema^c^
Skin prick, standardised extracts	Apply a drop, prick through with lancet.	15–20 min	Mean wheal diameter > 3 mm or ≥ 50% of histamine
Skin prick, non‐standardised extracts	Dissolve material, apply a drop, prick through with lancet.	15–20 min	Mean wheal diameter > 3 mm or ≥ 50% of histamine
Prick‐by‐prick	Prick solid material, use same lancet to prick skin.	15–20 min	Mean wheal diameter > 3 mm or ≥ 50% of histamine
Intradermal	Inject a small volume of sterile, diluted material intradermally using a 25‐gauge needle.	20–30 min	Wheal diameter ≥ 200% of initial papule and flare

^a^ Positive result criteria may differ in non‐immunologic (NI‐CoU) and immunologic (I‐CoU) contact urticaria; whealing is usually needed to diagnose I‐CoU [123].

^b^ Whealing can be graded/recorded on a scale of 0–3: 1 (slight, barely visible or palpable edema), 2 (unmistakable wheal) and 3 (wheal extending beyond the test area) [61].

^c^ In NI‐CoU, erythema readings are generally considered less informative than whealing readings [61]. Erythema can also be graded/recorded, for example, on a scale of 0–3: 1 (slightly perceptible erythema = slight pinkness), 2 (clearly visible erythema) and 3 (strong and spreading erythema) [23]. Erythema can be caused by solely irritant effects on the skin.

NI‐CoU is generally diagnosed based on medical history, including exposure history (e.g., photographs of cosmetic product ingredients and safety material data sheets), photographs of skin reactions, and positive non‐invasive skin tests (see Section 3.1 and Table 2).

For I‐CoU, IgE sensitisation must be confirmed using skin tests (e.g., SPTs, prick‐by‐prick tests), in vitro tests, or a combination of both. It is important to note that confirmed sensitisation does not alone indicate an allergic disease [13]. A diagnosis of IgE‐dependent allergy is based on a convincing clinical history and proven sensitisation to the culprit allergen (Table 2) [256]. Diagnosing I‐CoU caused by LMW chemicals can be challenging, as these substances are usually not commercially available for skin and in vivo tests.

NI‐CoU reactions may be suppressed by ultraviolet B (UVB) and ultraviolet A (UVA) irradiation [70], as well as by oral and topical nonsteroidal anti‐inflammatory drugs [16, 73, 257, 258, 259]. False‐negative reactions in I‐CoU may occur due to the use of H_1_‐antihistamines (if taken within five plasma half‐lives of drug elimination) [2], systemic glucocorticoids (if taken within 7 days) [2], tricyclic antidepressants (e.g., doxepin, amitriptyline) [260, 261, 262, 263, 264] or selective serotonin reuptake inhibitors (e.g., paroxetine, sertraline) [2, 263, 264].

### 3.1 Non‐Invasive Skin Tests

Non‐invasive skin tests measure skin reactivity and are used in the diagnosis of both NI‐CoU and I‐CoU. These tests are usually conducted in a stepwise manner, as outlined in Table 2. Each subsequent test is performed only if the previous one yields a negative result. However, to save time, multiple types of tests can be conducted concurrently. For I‐CoU diagnostics, some practitioners prefer to first perform SPTs and prick‐by‐prick tests due to time limitations and a generally lower risk of false‐negative results compared to non‐invasive skin tests [240].

In non‐invasive skin tests, fresh food and non‐edible plants can be used. Sourcing other materials for testing may be difficult. While many LMW triggers of CoU are commercially available for patch testing (marked as ‘PT’ in Table 3), these preparations are not standardised for evaluating CoU [265]. However, urticarial reactions may still be observed if patch tests used to assess type IV hypersensitivity are removed after 20 min [77]. Since I‐CoU can spread beyond the contact area, whealing may appear at the edges of patch tests even without their removal.

No universal vehicle for non‐invasive skin tests exists [72]. The most commonly used vehicles are water and petrolatum, but alcohol and the addition of propylene glycol can enhance the sensitivity of the test [16, 79]. In NI‐ICRs, both the vehicle and the concentration of the substance influence the strength of the reaction [62]. When testing materials of unknown irritancy, it is recommended to include at least five healthy, non‐allergic control subjects at the same stage where the positive result was obtained [21, 230, 239, 259, 266]. In cases of I‐CoU, control subjects typically test negative, whereas several controls may show a response in NI‐CoU [23].

#### 3.1.1 Open and Rub Test

The open test, also known as the open application test, is the simplest method for diagnosing NI‐CoU and I‐CoU [266]. However, it has not been evaluated in controlled trials, and standardised procedures are lacking [267]. Both LMW and HMW substances can be tested, with LMW testing being even less standardised. A sample of the suspected material (0.1 mL if liquid) is applied to a 3 × 3 cm area of skin. For multiple liquids, 10 μL is used on a 1 × 1 cm area [16]. In the rub test, the material is rubbed onto the skin 10 times with moderate pressure to enhance reactivity [160, 241]. These tests are typically performed on the upper back or extensor upper arm [1, 16, 21, 241], but they can also be conducted on the volar forearm [144, 148, 175, 268, 269]. If results are negative at the initial sites, testing may extend to slightly affected or previously involved areas [68, 185, 240], which may react more readily than healthy skin [259]. The face is often preferred for NI‐CoU testing due to its rich vascularisation, high permeability, and a relatively thin stratum corneum [16, 61, 69].

Results are usually assessed at 15–20, 40 and 60 min [26, 239, 241]. If initially negative, the substance may be reapplied [150]. A positive result is indicated by a persistent erythemato‐oedematous reaction [1, 21], though minimal reactions may appear solely as erythema [23, 62], which is less informative than whealing [61]. Minute papules, often observed, are believed to be small wheals rather than eczematous papules [270], and vesicles, a clear sign of acute eczema, are extremely rare in this test [8, 270].

High concentrations of allergens can induce anaphylaxis, though this is rare [271, 272]. If systemic symptoms are reported, testing should begin with serial tenfold dilutions (up to 1:10^6^) in saline solution (when soluble), and emergency care for anaphylaxis should be ensured (i.e., trained staff, administration of adrenaline, venous access, oxygen support, monitoring of vital signs etc.) [236]. Many allergens associated with CoU are commercially available for patch testing (marked with ‘PT’ in Table 3) but are not standardised for the open/rub test [171].

#### 3.1.2 Chamber Test

The chamber test, also known as the occlusive application test, enhances percutaneous penetration, potentially increasing sensitivity compared to the open test [16]. The substance is placed in a standard patch test chamber and moistened with saline solution if necessary. The chamber is usually applied to the skin on the upper back or the extensor side of the upper arm for 15–20 min [1, 16, 21, 259], though some practitioners conduct the test on the volar forearm [71, 196, 273, 274]. Adhesive tape is usually used to secure the chambers in place. For powdered substances, petrolatum may be applied to the chamber beforehand to improve adhesion [59]. The test site is evaluated 5 min after chamber removal [239] and again at two additional 20‐min intervals, with results interpreted similarly to those of the open test. In regular patch testing for type IV hypersensitivity (with a 2‐day occlusion), if patients experience severe pruritus shortly after chamber application, these areas should be further assessed, as an early urticarial reaction typically resolves before the next evaluation [185, 230].

#### 3.1.3 Repeated Open Application Test and Use Test

The repeated open application test and the use test may be needed to detect weak NI‐ICRs and delayed onset CoU [16, 275]. The former involves applying the suspected commercial product or special test substance twice daily to the volar side of the forearm near the antecubital fossa [246, 276], covering an area of at least one centimetre in diameter [277]. The size of the test area is usually between 3 × 3 cm and 5 × 5 cm [276]. In the diagnostic process of eczema, the test is conducted for up to 2 weeks, but signs of CoU are expected to appear sooner [275, 278]. In the use test, the suspected product or substance is applied to a previously clinically involved anatomic site in the same manner as it was used before [16].

### 3.2 Invasive Skin Tests

Invasive skin tests are used in the diagnostic process for type I allergies [266, 271]. They are performed only on normally appearing skin, typically on the volar forearm. It is essential to include both positive (histamine dihydrochloride, 10 mg/mL) and negative (saline solution, 0.9% NaCl) controls [44, 259]. While SPTs are relatively standardised, most other tests lack standardisation. Test concentrations must be carefully chosen to avoid nonspecific reactions. For materials of unknown irritancy, control subjects should be tested, as with non‐invasive skin tests.

#### 3.2.1 Skin Prick Tests With Standardised Allergen Extracts

SPTs with standardised, commercially available allergen extracts have established sensitivity and specificity [267], making them an important tool in the investigation of I‐CoU and PCD [241]. SPTs are easy to perform and reproducible. They are considered safer than both the scratch and intradermal tests, as they involve introducing a smaller dose of the allergen into the skin.

SPTs are typically conducted using a series of pricks. A drop of allergen extract is placed on the skin, and the skin is punctured through the drop with a lancet needle, bringing the substances into contact with skin mast cells [266]. Drops need to be spaced at least 2 cm apart to prevent the spread of wheals from one positive test site to adjacent tested areas, which could complicate the interpretation of results.

After pricking the skin, the drops are wiped off with a soft tissue. The wheal size is determined 15–20 min after application by measuring the largest perpendicular diameters. The result is the sum of these diameters divided by two. Wheals larger than 3 mm and at least half the size of the wheal produced by the positive control are regarded as positive [259, 267, 271]. Wheals smaller than half the size of the positive control are usually considered not significant [259]. If a measurable reaction occurs to the negative control, the SPT is generally regarded as inconclusive, and re‐testing may be necessary. In such cases, patients are usually further evaluated using in vitro techniques. Some define positive SPTs as having a mean wheal diameter at least 3 mm larger than that of the negative control [152].

Since SPTs evaluate only sensitisation and not clinical disease, positive reactions must always be correlated with the patient's history, including exposure [267]. Evaluation for cross‐sensitisation (e.g., shrimp and house dust mite, fresh apple peel and birch pollen) should also be performed if suspected based on the history to identify the primary sensitiser [267].

Testing with commercially available food allergens often results in false negatives, possibly due to incomplete extraction and/or denaturation of labile allergens. Additionally, standardised allergen dilutions for many allergens causing I‐CoU are not available [241, 267]. In such cases, SPTs using non‐standardised extracts and/or prick‐by‐prick tests can be performed.

#### 3.2.2 Skin Prick Tests With Non‐standardised Allergen Extracts

SPTs with self‐made test materials are less commonly used to diagnose I‐CoU. Since nearly all allergens that cause type I hypersensitivity are water‐soluble, adding a small amount of saline solution is usually sufficient to dissolve dry raw material (e.g., flour) [279]. For LMW allergens that cause I‐CoU, a solution of hapten and human serum albumin conjugate can be prepared, although this may not be readily available [13, 16, 280, 281]. It is advisable to conduct SPTs with non‐standardised allergen extracts in duplicate.

#### 3.2.3 Prick‐by‐Prick Tests

Prick‐by‐prick tests, a variant of SPTs, are specifically designed for non‐standardised, particularly solid allergen sources (e.g., fresh foods). Plants usually contain sufficient water to enable the transfer of allergenic material to the skin. Typically, a standard panel of the most relevant local allergens is used. Many of these allergens can be kept frozen in separate compartments and defrosted before testing. Additional testing with materials relevant to the patient's case history (e.g., foods, plants, drugs, chemicals) is often conducted [267]. The material is first pricked with the lancet, and then the skin is immediately punctured with the same lancet [241, 266]. The reaction is assessed after 15–20 min, as described for SPTs [241, 266].

#### 3.2.4 Scratch Test

The scratch test is not recommended for routine use, as it offers no clear advantages over SPTs [267]. It can, however, be used if SPTs and/or prick‐by‐prick tests are negative and/or only for non‐standardised allergens are available [1, 21, 239, 259, 266]. Compared to SPTs, the scratch test is less standardised (e.g., producing consistent scratches is challenging), has a higher risk of false‐positive reactions (e.g., nonspecific reactions that must be considered when interpreting results), and lacks sensitivity [266, 282]. There is believed to be a lower chance of skin infections than in SPTs, which is important in SPTs with meats for example [161, 259]. A survey involving 133 members of the American Contact Dermatitis Society found that only 19% performed scratch testing in patients with suspected CoU [265].

The skin barrier is first carefully broken by a 5 mm long linear scratch with a venipuncture needle or SPT lancet, without inducing bleeding [241, 259]. The substance is then applied directly to the scratched skin, left in place for 15–20 min, and the test is subsequently read [1]. Allergen solutions are usually wiped off with a soft tissue after 5–10 min [259] and the results are also read 15–20 min after application [259]. Only the largest diameter of the wheal perpendicular to the scratch is measured [259]. A positive reaction is defined as a wheal diameter that is equal to or greater than that produced by histamine [241, 259].

#### 3.2.5 Scratch‐Chamber Test

Similar to the scratch test, the scratch‐chamber test is also not recommended for routine use. However, because it can detect both type I and type IV hypersensitivity, it may be used in the diagnostic workup of PCD [267, 281]. This method allows the penetration of HMW protein allergens, which typically cannot easily penetrate the intact horny layer of the epidermis [267, 283]. The skin barrier is broken by a 5‐mm long linear scratch, as previously described. The substance is then put into an ordinary patch/chamber, which is fixed directly onto the scratched skin for 15–20 min using a porous tape [1, 16, 21]. The test is first read 5 min after removing the patch/chamber [239], during which urticarial reactions may be detected. The control substances used for this test are the same as those for other invasive skin tests [259]. To assess delayed reactions, the patch or chamber is reapplied to the skin, removed after 24 h, and read a second time [284]. Additional readings can be performed on Day 2 and Day 4 [284].

#### 3.2.6 Intradermal Test

Intradermal tests are rarely used in the diagnostic process of I‐CoU. They are typically reserved for selected cases that are negative on SPTs, particularly when drug allergy is suspected. A small volume of sterile, diluted allergen is injected intradermally using a 25‐gauge needle. A small raised papule should appear, and it must be outlined with a pen. The wheal develops over a period of 20–30 min and should also be outlined. The test is typically considered positive when the diameter of the wheal is twice the size of the initial papule and is surrounded by a typical flare [267]. Intradermal tests have high sensitivity but lower specificity, and they are technically more challenging to perform and interpret in a standardised and reproducible manner compared to SPTs. Additionally, these tests are more painful and carry a higher risk of systemic reactions [267]. Therefore, they can only be conducted in facilities equipped with resuscitation equipment.

### 3.3 In Vitro Techniques

#### 3.3.1 Antigen‐Specific Serum IgE Antibodies

In vitro techniques for measuring the level of serum IgE antibodies to specific suspected allergen sources are useful in the work‐up of suspected I‐CoU, but are available for only a limited number of allergens. The CAP technique (ImmunoCAP Test, Thermo Fisher) is the most commonly used [2, 16]. Currently, there is also a large number of recombinant allergens available on the market, enabling testing for the presence of IgE specific for individual allergen proteins in the so‐called component‐resolved diagnosis. Such in vitro tests can be performed by investigating the suspect allergens individually using singleplex ImmunoCAP, particularly when there is a specific allergen source in question (e.g., I‐CoU in association with lipid transfer protein). Alternatively, specific IgE can be measured for numerous allergens from different origins simultaneously on the same platform using multiplex testing (e.g., 112 allergen components from 48 different allergen sources in ImmunoCAP ISAC 112 test, Thermo Fisher), sometimes in conjunction with full allergenic extracts (ALEX [Allergy Explorer] test; Macroarray Diagnostics). The ISAC platform includes only recombinant and some natural allergen molecules, whereas ALEX includes both allergen molecules and full extracts of allergen sources. These tests can also be used for the assessment of potential cross‐reactivity between allergens [1]. For example, patients allergic to NLR often show cross‐reactivity with homologous allergens in fruits (more rarely vegetables), a condition sometimes called ‘latex‐fruit syndrome’ [1].

#### 3.3.2 Basophil Activation Test

The basophil activation test is a flow cytometric assay that detects the functional ability of IgE to activate basophils when stimulated with an allergen. Basophil activation is measured by flow cytometry, detecting the expression of activation‐associated molecules (e.g., CD63) on the cell membrane after exposure to an allergen [198, 266, 285]. The basophil activation test requires basophils from healthy donors, patient blood and the relevant allergen in its natural form. Routine clinical applications of this test are not yet feasible due to a lack of full standardisation [285] and it is more commonly available in tertiary centres with specialised laboratories.

#### 3.3.3 Histamine Release Test

The histamine release test can also be used to confirm an IgE‐mediated mechanism for allergy. In this test, basophils are incubated with the patient's serum. If specific IgE antibodies are present, the basophils can become passively sensitised. These sensitised cells are then exposed to various concentrations of an allergen (e.g., chlorhexidine). Histamine release, reflecting basophil activation, is quantitatively measured [102, 215].

## 4 Differential Diagnosis

When diagnosing I‐CoU and NI‐CoU, it is essential to consider all other forms of chronic or acute recurrent urticaria. Dermographism should be excluded. Wheals caused by NI‐CoU (e.g., from substances like benzoic and sorbic acid) after showering may resemble cholinergic urticaria. In cases of facial swelling, it is also important to consider type IV‐induced ‘airborne contact edema’ from substances such as isothiazolinones or acrylates, as well as allergic contact dermatitis from hair dyes [246]. Wheals in CoU do not blister and there are no residual skin changes like scaling or pigmentation. However, scaling or persistent eczema may occur in PCD.

In cases of PCD, other eczematous conditions, such as allergic and irritant contact dermatitis, atopic dermatitis, dyshidrotic eczema and photoallergic or phototoxic contact dermatitis must be considered [246]. Therefore, additional tests like patch testing or photo‐patch testing are often required [1, 246, 286]. In chronic eczema resulting from PCD, the dermatitis usually resolves more readily once the trigger(s) is/are removed, compared to ‘regular’ chronic hand eczema.

## 5 Management

### 5.1 Trigger Identification and Avoidance

CoU can be prevented and successfully treated if correctly diagnosed [21]. The most important steps are early trigger identification and complete avoidance of the culprit, especially in cases of I‐CoU [287]. Occupational I‐CoU should be managed by eliminating the allergen from the direct work environment. If it is not possible to remove the trigger, other measures are required to reduce allergen exposure, such as using appropriate gloves and cotton liners [1, 2, 238]. In cases of NI‐CoU, where the mechanism is non‐allergic and more dose‐dependent, strict avoidance may not always be necessary (e.g., in cases of sorbic acid in cosmetics). However, decreasing exposure frequency and dose may help alleviate or even eliminate signs and symptoms. Patients working in the food industry, for example, may be exposed to multiple allergens [236]. Collaboration with occupational health services will facilitate the implementation of avoidance strategies and other safety and preventive measures in the workplace [288]. If avoidance of further contact with causative substances is not entirely feasible and CoU impacts the patient's quality of life, pharmacological agents for symptomatic relief and prevention may be considered.

### 5.2 Treatment

The choice of treatment may vary depending on the type of reaction and its pathophysiology [287]. In I‐CoU, second‐generation H_1_‐antihistamines (sgAHs) at up to four times the licensed daily dose are the first‐line treatment, as histamine release plays a key role in its pathogenesis [1, 239]. In severe I‐CoU cases, with or without related PCD, where allergen avoidance is not possible or patients are refractory to sgAHs, anti‐IgE therapy with omalizumab may be beneficial [1, 289]. SgAHs and nonsteroidal anti‐inflammatory drugs are usually not effective for NI‐CoU [1, 239]. In PCD, topical corticosteroids can reduce local inflammation, while systemic glucocorticoids are typically reserved for the initial stages of severe cases. In severe PCD, other systemic therapies (e.g., cyclosporine, methotrexate) may be considered [290]. The use of newer biologics, such as dupilumab, requires further research, particularly for PCD, as it has shown efficacy in chronic hand eczema in patients with atopic dermatitis [290]. However, dupilumab did not benefit an isolated case of I‐CoU [27]. Patients at increased risk for anaphylaxis should also carry an adrenaline auto‐injector at all times and wear a medical alert bracelet.

## 6 Conclusion

CoU remains underrepresented in guidelines and publications on (chronic) urticaria. CoU and related conditions continue to present significant challenges in diagnosis and management, as they can manifest concurrently as different aspects of the CoU syndrome. Interdisciplinary collaboration among dermatologists, allergists and occupational physicians is often necessary and beneficial for achieving optimal outcomes.

## Author Contributions

**Mojca Bizjak:** conceptualization, methodology, investigation, project administration, supervision, writing – original draft, visualization, data curation, validation, formal analysis, resources. **Olivier Aerts:** conceptualization, methodology, writing – review and editing, validation, data curation. **David Pesqué:** conceptualization, methodology, writing – review and editing. **Melba Muñoz:** conceptualization, methodology, writing – review and editing. **Riccardo Asero:** conceptualization, methodology, writing – review and editing. **Margarida Gonçalo:** conceptualization, methodology, writing – review and editing, validation. **Thomas Rustemeyer:** conceptualization, methodology, writing – review and editing. **Mitja Košnik:** conceptualization, methodology, funding acquisition, writing – review and editing. **Mark Kačar:** conceptualization, methodology, writing – review and editing. **An Goossens:** conceptualization, methodology, writing – review and editing. **Jose Hernán Alfonso:** conceptualization, methodology, writing – review and editing. **Charlotte G. Mortz:** conceptualization, methodology, writing – review and editing. **Maryam Ali Al‐Nesf:** conceptualization, methodology, writing – review and editing. **Joachim W. Fluhr:** conceptualization, methodology, writing – review and editing. **Howard I. Maibach:** conceptualization, methodology, writing – review and editing. **Ana Maria Giménez‐Arnau:** conceptualization, methodology, supervision, writing – review and editing, validation.

## Ethics Statement

The authors have nothing to report.

## Conflicts of Interest

The authors declare no conflicts of interest.

## Supporting information


**Table S1.** Chemical Abstracts Service registry (CAS) numbers of triggers of contact urticaria and related conditions listed in Table 3.

## Data Availability

The data that support the findings of this study are available on request from the corresponding author. The data are not publicly available due to privacy or ethical restrictions.
