# Supplementary material for: Contact Urticaria and Related Conditions: Clinical Review
Source: Contact Dermatitis. 2025 Apr 2;93(2):87–107. doi: 10.1111/cod.14794 (PMC12223959; doi:10.1111/cod.14794)
Supplement: Supplementary file 1 — Table S1. Chemical Abstracts Service registry (CAS) numbers of triggers of contact urticaria and related conditions listed in Table 3. [file COD-93-87-s001.docx]

**Table S1.** Chemical Abstracts Service registry (CAS) numbers of triggers of contact urticaria and related conditions listed in **Table 3**.

| Trigger | CAS no. |
| --- | --- |
| Algae | / |
| *p*-Aminophenol^†PT^ | 123-30-8 |
| Ammonium persulfate^†PT^ | 7727-54-0 |
| α-amylase^CAP^ | 9000-90-2 |
| Apple^CAP,SPT^ | / |
| Asparagus^CAP^ | / |
| Bacitracin^PT^ | 405-87-4 |
| Balsam of Peru *(Myroxylon pereirae)*^†PT^ | / |
| Benzaldehyde^†PT^ | 100-52-7 |
| Benzoic acid (B.A.)^†PT^ | 65-85-0 |
| Benzophenone-3^†PT^ (oxybenzone) | 131-57-7 |
| Bisphenol A epoxy resin^PT^ | 25068-38-6 |
| Cannabis^†^ | 8063-14-7 |
| Capsaicin^†^ | 404-86-4 |
| Carrot^CAP,SPT^ | / |
| Carvone^†^ | 6485-40-1 |
| Caterpillars | / |
| Cellulase | 9012-54-8 |
| Cefotiam | 66309-69-1 |
| Chamomile (*Chamomilla recutita*^†^) | 84082-60-0 |
| Chlorhexidine^†PT,CAP^ | 55-56-1 |
| Chlorocresol (*p*-Chloro-m-cresol^†^)^PT^ | 59-50-7 |
| Chromium (potassium dichromate^PT^) | 7778-50-9 |
| Chrysanthemum^SPT^ | / |
| Cinnamic acid (C.A.)^†^ | 621-82-9 |
| Cinnamic aldehyde^†^ (cinnamal)^PT^ | 104-55-2 |
| Cinnamomum cassia oil^†^ | 84961-46-6 |
| Cobalt (cobalt chloride^PT^) | 7440-48-4 |
| Colophonium^†PT^ | 8050-09-7 |
| Copper^PT^ | 7440-50-8 |
| Coumarin^†PT^ | 91-64-5 |
| Cow’s dander^CAP,SPT^ | / |
| Cow’s milk^CAP,SPT^ | / |
| Dimethyl sulfoxide (DMSO) | 67-68-5 |
| Diphenylmethane-diisocyanate (MDI) | 101-68-8 |
| Egg^CAP^ | / |
| Equae Lac^†^ (mare’s milk^CAP^) | / |
| Ethyl alcohol^†^ (ethanol) | 103-11-7 |
| 2-ethylhexyl acrylate^†PT^ | / |
| Eugenol^†PT^ | 97-53-0 |
| *Ficus benjamina*^CAP^ | / |
| Fishing baits (worms) | / |
| Formaldehyde^†PT,CAP^ | 50-00-0 |
| Garlic^CAP,SPT^ | / |
| Geraniol^†PT^ | 106-24-1 |
| Glycolic acid^†^ | 79-14-1 |
| Honey (Mel^†^)^CAP^ | 8028-66-8 |
| Hydolysed animal proteins (hydrolysed collagen^†^, hydrolysed milk protein^†^) | 92113-31-0 |
| Hydrolysed wheat protein^†^ | 70084-87-6 |
| Jellyfish venom | / |
| Kiwi^CAP^ | / |
| Lanolin^†^ alcohol^PT^ | 8006-54-0 |
| Lilies (*Lilium*) | / |
| Mango^CAP,SPT^ | / |
| Meat, beef^CAP,SPT^ | / |
| Meat, pork^CAP,SPT^ | / |

**Table S1.** *Cont.*

| Trigger | CAS no. |
| --- | --- |
| Meat, chicken^CAP,SPT^ | / |
| Menthol^†PT^; Peppermint plant (*Mentha piperita*)^CAP^ | 89-78-1 |
| Methylisothiazolinone^†^/methylchloroisothiazolinone^†PT^ | 2682-20-4 |
| Natural rubber latex (NRL)^CAP,SPT^ | 9006-04-6 |
| Neomycin (neomycin sulfate^PT^) | 1404-04-2 |
| Nickel (nickel sulfate^PT^) | 7440-02-0 |
| Nicotinic acid (niacin) esters (e.g., methyl nicotinate^†^) | 93-60-7 etc. |
| Oat (*Avena sativa*^†^)^CAP,SPT^ | 84012-26-0 |
| Onion^CAP,SPT^ | / |
| Panthenol^†PT^ | 16485-10-2 |
| Papain^CAP^ | 9001-73-4 |
| Paraben(s)^† PT^ | 94-26-8 etc |
| Paratoluenediamine (PTD, toluene-2,5-diamine sulfate^†^)^PT^ | 615-50-9 |
| Peach^CAP,SPT^ | / |
| Phenoxyethanol^†PT^ | 122-99-6 |
| *p*-Phenylenediamine (PPD)^†PT^ | / |
| Polyhexanide (polyaminopropyl biguanide^†^)^PT,CAP^ | 133029-32-0 |
| Polymyxin B^PT^ | 1404-26-8 |
| Potato^CAP,SPT^ | / |
| Potassium persulfate^†^ | 7727-21-1 |
| Povidone (PVP^†^, polyvinylpyrrolidone)^PT^ | 9003-39-8 |
| Povidone iodine^PT^ | 25655-41-8 |
| Pramoxine^PT^ | 140-65-8 |
| Protease | 39450-01-6 |
| Rice^CAP,SPT^ | / |
| Runner bean (*Phaseolus multiflorus*) | / |
| Rye^CAP,SPT^ | / |
| Salmon^CAP,SPT^ | / |
| Seafood (i.e., fish, crustaceans)^CAP,SPT^ | / |
| Sesame *(Sesamum indicum*^†^)^CAP,SPT^ | 8008-74-0 |
| Silk^CAP^ | / |
| Sodium benzoate^†PT^ | 532-32-1 |
| Sodium hypochlorite | 7681-52-9 |
| Sorbic acid (S.A.)^†PT^ | 22500-92-1 |
| Soybean^CAP,SPT^ | / |
| Spider mite (*Tetranychus urticae*) | / |
| Spices | / |
| Stinging nettle (*Urtica dioica*)^CAP,SPT^ | / |
| Storage mites^CAP^ | / |
| Streptomycin^PT^ | 57-92-1 |
| Tomato^CAP,SPT^ | / |
| Triclosan^†PT^ | 338034-5 |
| Tulip | / |
| Turpentine^†PT^ | 9005-90-7 |
| Vanillin^†PT^ | 121-33-5 |
| Walnut^CAP,SPT^ | / |
| Wheat^CAP,SPT^ | / |
| Wool (ewe) | / |
| Xylanase (hemicellulase) | 9025-57-4 |

Note: Low molecular weight chemicals (<1000 Daltons [g/mol]) are marked in **bold**.

^†^INCI (International Nomenclature of Cosmetic Ingredients) name.

^CAP^Commercially available for CAP testing.

^PT^Commercially available for patch testing.

^SPT^Commercially available for skin prick testing.
